# Topical Delivery of Curcumin by Choline-Calix[4]arene-Based Nanohydrogel Improves Its Therapeutic Effect on a Psoriasis Mouse Model

**DOI:** 10.3390/ijms21145053

**Published:** 2020-07-17

**Authors:** Alessia Filippone, Grazia M. L. Consoli, Giuseppe Granata, Giovanna Casili, Marika Lanza, Alessio Ardizzone, Salvatore Cuzzocrea, Emanuela Esposito, Irene Paterniti

**Affiliations:** 1 Department of Chemical, Biological, Pharmaceutical and Environmental Sciences, University of Messina, Viale Ferdinando Stagno D’Alcontres, 31-98166 Messina, Italy; afilippone@unime.it (A.F.); gcasili@unime.it (G.C.); mlanza@unime.it (M.L.); aleardizzone@unime.it (A.A.); salvator@unime.it (S.C.); eesposito@unime.it (E.E.); 2 Institute of Biomolecular Chemistry-C.N.R., Via P. Gaifami 18, 95126 Catania, Italy; grazia.consoli@icb.cnr.it (G.M.L.C.); giuseppe.granata@icb.cnr.it (G.G.)

**Keywords:** psoriasis, inflammation, imiquimod, curcumin, calixarene

## Abstract

Curcumin (CUR) has shown remarkable efficacy in the treatment of skin diseases, but its effective transdermal delivery is still a major challenge and stimulates interest in the design of novel systems for CUR dispersion, preservation, and delivery facilitation to the deeper layers of the skin. The present work aimed to investigate the potential of a nanohydrogel, formed by a micellar choline-calix[4]arene amphiphile (CALIX) and CUR, in the treatment of skin diseases through an imiquimod (IMQ)-induced psoriasis model. Psoriasis plaques are associated with aberrant keratinization, abnormal distribution of tight junctions (TJs) proteins, and enhanced expression of inflammatory markers. The nanohydrogel restored the normal distribution of TJs proteins ZO1 and occludin and reduced the expression of TNF-α and inducible nitric oxide synthetase (iNOS) compared to the untreated IMQ group. The novelty lies in the calix[4]arene-based nanohydrogel as a potential new soft material for the topical skin delivery of CUR. The nanohydrogel, due to its physicochemical and mechanical properties, enhances the drug water-solubility, preserves CUR from rapid degradation, and eases the local skin administration and penetration.

## 1. Introduction

The impact of psoriasis, as an inflammatory skin cell-mediated disease supported by a hyper-activation of cutaneous and immune cells, is remarkable on the world population, involving people of different ethnicities and ages [1]. Psoriasis is characterized by an abnormal epidermal proliferation, aberrant differentiation of keratinocytes, thick inflammatory cell infiltrates, and abnormal dermal angiogenesis. The psoriasis pathogenesis is complex and varied, for which it became necessary to deepen multi-targets therapeutics interventions. Currently, the therapeutic agents such as anti-inflammatory and immunomodulators are available for the treatment of psoriasis as topical or systemic therapy, but they have many side effects such as cutaneous atrophy and rebound of the disease. Thus, there remains a need to have more treatment options and more safe alternatives.

Herbal natural drugs appear a more safe, effective, and affordable alternative to conventional therapeutics [2,3,4].

Curcumin (CUR), an active ingredient derived from the Rizoma of Indian turmeric spice (*Curcuma longa*), has shown a wide range of medicinal properties and multiple health benefits [5], most related to its anti-proliferative, anti-inflammatory, antioxidant, and immunomodulatory activities. CUR has also demonstrated effective activity in the treatment of psoriasis with the absence of obvious side effects [6]. The anti-psoriasis effect of CUR can be related to different mechanisms of action including reduction of the oxidative stress and down-regulation of pro-inflammatory cytokines with consequent inhibition of nuclear factor NF-κB and enhancement of the skin barrier function [7,8].

In the treatment of inflammatory skin diseases, topical administration is the most important approach compared to oral and injection ones. The drug applied to the affected skin can directly work on the inflammatory region to improve the symptoms, reducing adverse systemic effects. Unfortunately, the quantity of drug percutaneously absorbed is slowed down and diminished by the corneum stratum. To overcome this drawback, nanotechnology is proving a promising strategy, and topical nanostructured drug delivery systems have been successfully developed for the treatment of skin diseases, including psoriasis [9]. The nano-formulation of CUR has resulted in enhanced drug local concentration, solubility, stability, and skin penetration [10], and in an improvement of the drug effectiveness in psoriatic mice [11,12,13,14]. In the search for novel building blocks to realize nanostructured drug delivery systems, calix[n]arene macrocycles are attractive molecular scaffolds [11,12]. The calix[n]arene skeleton can be variously functionalized to give amphiphilic derivatives capable to assemble in micelles, vesicles, solid lipidic nanoparticles, and gels [13] of interest for biomedical and pharmaceutical applications [15].

Recently, we reported that a polycationic amphiphilic choline-calix[4]arene derivative **1** (CALIX) spontaneously self-assembles in micellar nanoaggregates capable of loading CUR and delivering it in the mouse eye when administered as an eye drop [16]. At higher concentrations of CALIX and CUR, the micelles organize in a supramolecular nanohydrogel (CALIX/CUR), in a biomimetic medium like 10 mM PBS, pH 7.4 [17]. The CALIX/CUR nanohydrogel formed by biofriendly components exhibited viscoelasticity, self-healing, injectability, ease skin application, and skin absorption that suggested it promising as a novel soft material for the topical administration of CUR.

Among the nano-formulations, hydrogels offer the advantages to improve skin hydration and provide protracted permanence in the affected skin area, prolonged and sustained release of the drug with consequent reduction of the dose and administration frequency. Thus, in this paper, we decided to investigate the anti-psoriasis effect of the CALIX/CUR nanohydrogel in an in vivo model of psoriasis induced by imiquimod (IMQ) that produces a psoriatic plaque model. Inflammatory markers, that are typical in psoriasis, like TNF-α, IL1β, and mast cell degranulation, as well as occludin and ZO-1 tight junctions (TJs), were examined.

## 2. Results

### 2.1. CALIX/CUR Hydrogel

**Figure 1:**
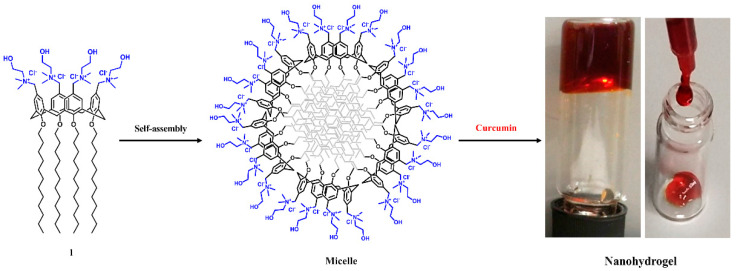
Preparation of micellar choline-calix[4]arene amphiphile/curcumin (CALIX/CUR) hydrogel. Structure of the CALIX (1); schematic representation of the CALIX **1** micelle, and photographs of the CALIX/CUR nanohydrogel.

CALIX bearing dodecyl alkyl chains at the lower rim and choline polar ligands at the upper rim (Figure 1), spontaneously self-assembles in micellar nanoaggregates (46 nm diameter) at low concentration (critical micellar concentration 8.5 µM) in 10 mM PBS medium [18].

The nanoaggregates are capable of solubilizing CUR in PBS medium to give a micellar colloidal solution [16] or a micellar nanohydrogel (Figure 1) [17] under stimulus concentration. The viscoelastic, injectable, spreadable, and self-healing CALIX/CUR nanohydrogel showed mechanical properties suitable for topical skin application. The nanohydrogel slowly dissolves in PBS medium (0.30 ± 0.02%/h × mm of hydrogel thickness), releasing CUR-loaded micellar nanoaggregates (90 nm mean hydrodynamic diameter, 23 mV surface zeta potential) [17] as CUR delivery systems. The entrapment in micellar nanoaggregates is beneficial to maintain CUR solubility and stability, which are known to be poor in PBS medium [19].

### 2.2. Effects of Hydrogel Treatment on Psoriasis-Like Histological Damage in IMQ-Treated BALB/c Mice

**Figure 2:**
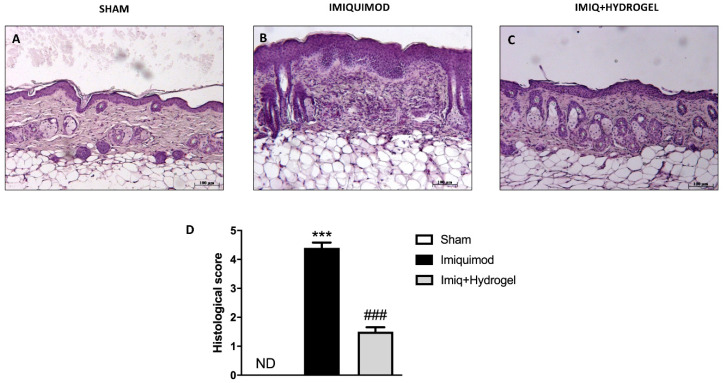
Effects of Hydrogel treatment on psoriasis-like histological damage in imiquimod (IMQ)-treated BALB/c mice. Histological analysis and histological score of right flank skin. No histological damage was detected in the analysis of the sham group slides (**A**, magnification 10×), see histological score (**D**). The IMQ-treated group presented significant histological damage accompanied by inflammatory cell infiltration (**B**, magnification 10×) see histological score (**D**). Hydrogel treatment decreased the accentuation of the inflammatory condition and epidermal thickening due to the IMQ treatment (**C**, magnification 10×), see histological score (**D**). Data are representative of at least three independent experiments; One-Way ANOVA test. *** *p* < 0.001 vs. sham; ^###^ *p* < 0.001 vs. IMQ. ND not detectable.

Histopathology of psoriatic skin (IMQ group) is characterized by hyperkeratosis (thickening of stratum corneum), parakeratosis (retention of nuclei in stratum corneum), acanthosis (increased thickness of malpighian layer), and epidermal infiltrates. CALIX /CUR hydrogel group showed very similar histopathological characteristics to that of the normal group, where epidermis was almost normalized, and less infiltrates were observed. H/E staining was performed to examine the structural differences in the tissues of mice from each experimental group. The control group did not show significant morphological alterations. The histological staining showed regular epidermis and dermis (Figure 2A, see histological score Figure 2D). Histopathology of the IMQ-treated group (Figure 2B, see histological score Figure 2D) was characterized by hyperkeratosis (thickening of stratum corneum), parakeratosis (retention of nuclei in stratum corneum), acanthosis (increased thickness of malpighian layer), and epidermal infiltrates. Instead, the hydrogel-treated group showed an important difference compared to the IMQ group. Mice treated with CALIX /CUR hydrogel group showed very similar histopathological characteristics to that of the normal group, where epidermis was almost normalized, and less infiltrates were observed (Figure 2C, see histological score Figure 2D).

### 2.3. Effect of Hydrogel Treatment on TJs Proteins 

**Figure 3:**
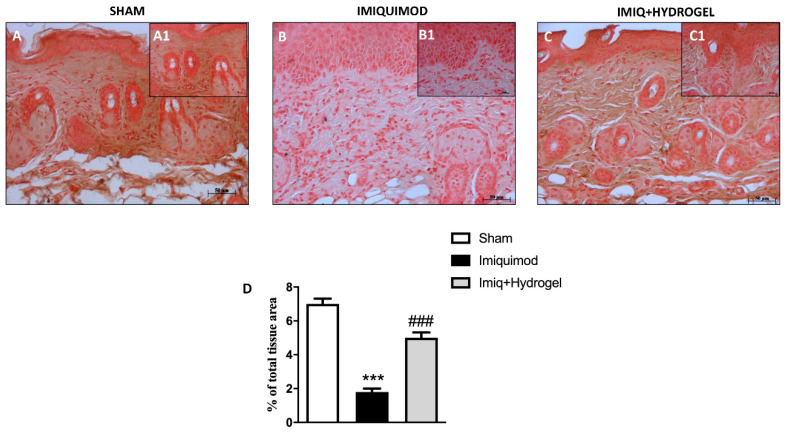
Effect of Hydrogel treatment on ZO-1 expression. The ZO-1 expression was analyzed using immunohistochemistry. Basal expression of ZO-1 (**A** magnification 20×, **A1** magnification 40×, **D**) has been found in the tissues of sham-operated mice, IMQ administration reduced this expression (**B** magnification 20×, **B1** magnification 40×, **D**), while Hydrogel treatment restored the expression of ZO-1 to almost basal levels (**C** magnification 20×, **C1** magnification 40×, **D**). Data are representative of at least three independent experiments; One-Way ANOVA test. *** *p* < 0.001 vs. sham; ^###^ *p* < 0.001 vs. IMQ.

**Figure 4:**
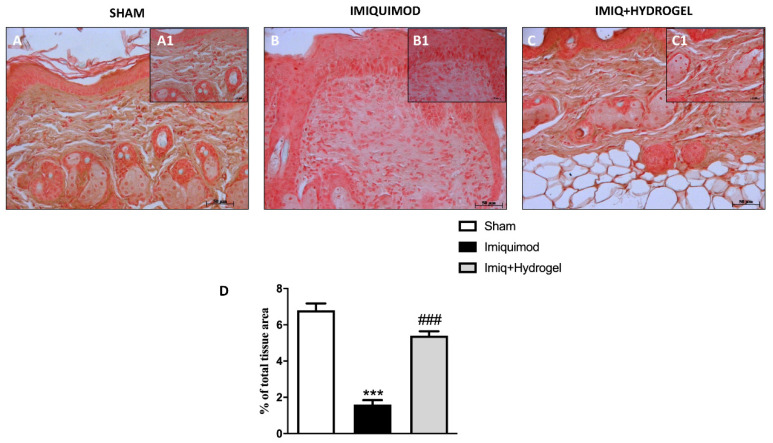
Effect of Hydrogel treatment on Occludin expression. The Occludin expression was analyzed by immunohistochemistry. Endogenous expression of Occludin has been found in the slides of sham-operated mice (**A** magnification 20×, **A1** magnification 40×, **D**), IMQ administration reduced this expression (**B** magnification 20×, **B1** magnification 40×, **D**). However, Hydrogel treatment increased the positive staining to Occludin (**C** magnification 20×, **C1** magnification 40× **D**). Data are representative of at least three independent experiments; One-Way ANOVA test. *** *p* < 0.001 vs. sham; ^###^ *p* < 0.001 vs. IMQ.

TJs like Occludin and ZO-1 are essential for the barrier function of the skin, guaranteeing a certain degree of occlusion and the variation of cell permeability [20]. Thus we investigated, by immunohistochemical analysis, the expressions of ZO-1 and Occludin, and observed a basal expression of both ZO-1 (Figure 3A, A1, see histological score Figure 3D) and Occludin (Figure 4A, A1, see histological score Figure 4D) in the tissue of sham-operated animals. These expressions, on the other hand, were significantly decreased in the IMQ-treated group (Figure 3B, B1, see histological score Figure 3D and Figure 4B, B1, see histological score Figure 4D), indicating that the induction of psoriasis causes greater disruption of the TJs, thus affecting cellular permeability. CALIX/CUR hydrogel treatment could considerably restore the expressions of both ZO-1 (Figure 3C, C1, see histological score Figure 3D) and Occludin (Figure 4C, C1, see histological score Figure 4D) almost to the levels of the sham group, thus contributing to the re-establishment of selective permeability.

### 2.4. Effect of Hydrogel Treatment on Mast Cell Quantification

**Figure 5:**
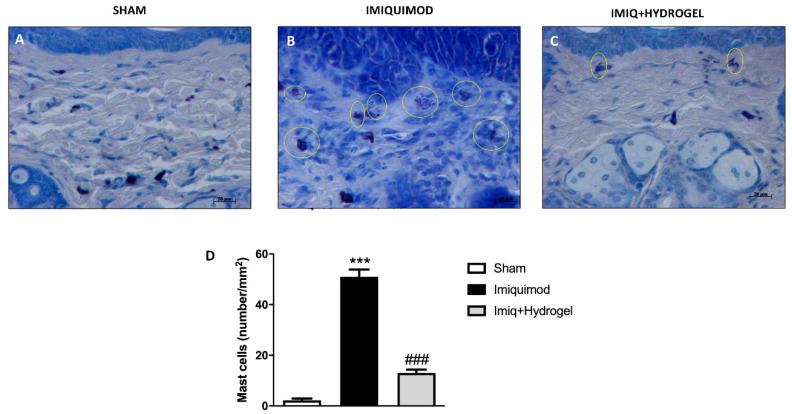
Effect of Hydrogel treatment on Mast cell quantification. Mast cell counts by toluidine blue staining. An increased number of mast cells were identified in the epidermis of IMQ-treated mice (**B** magnification 40×, **D**), compared to the control group (**A** magnification 40×, **D**). Hydrogel administration reduced the presence of mast cell infiltrate (**C** magnification 40×, **D**). Data are representative of at least three independent experiments; One-Way ANOVA test. *** *p* < 0.001 vs. sham; ^###^ *p* < 0.001 vs. IMQ.

Since the immune system plays a crucial role in the etiopathogenesis of psoriasis, we investigated through toluidine blue staining, mast cell infiltration, and degranulation [21]. The epidermal tissue of the sham-operated group did not show an evident inflammatory state—this is confirmed by the absence of mast cell infiltration (Figure 5A, see histological score Figure 5D). Conversely, treatment with IMQ caused an increased mast cell degranulation in the lesioned right flank (Figure 5B, see histological score Figure 5D). While, mice treated with CALIX /CUR hydrogel showed a significant reduction in the number of mast cells and their degranulation (Figure 5C, see histological score Figure 5D).

### 2.5. Effect of Hydrogel Treatment on Pro-Inflammatory Cytokines

**Figure 6:**
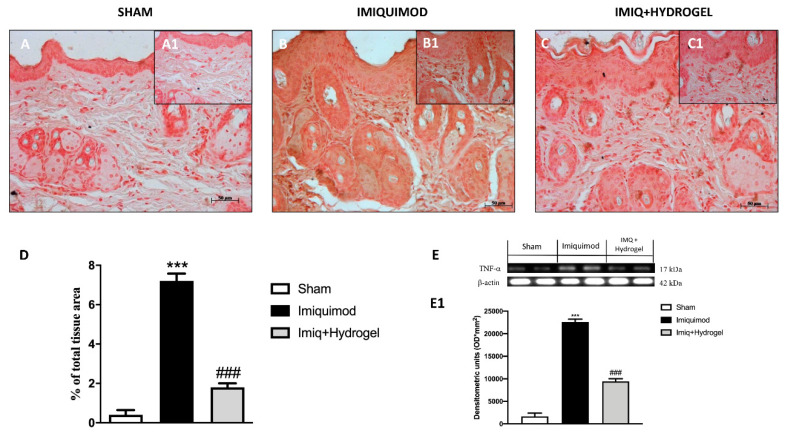
Effect of Hydrogel treatment on TNF-α expression. Immunohistochemical analysis of TNF-α revealed a significantly increased expression in the vehicle group (**B** magnification 20×, **B1** magnification 40× **D**,**E,E1**) compared to the control group (**A**, **B** magnification 20×, **A1** magnification 40×, **D**,**E**,**E1**). Hydrogel treatment reduced the expression of TNF-α in animals exposed to IMQ damage (**C**,**B** magnification 20×, **C1** magnification 40×, **D**,**E**,**E1**). Data are representative of at least three independent experiments; One-Way ANOVA test. *** *p* < 0.001 vs. sham; ^###^ *p* < 0.001 vs. IMQ.

**Figure 7:**
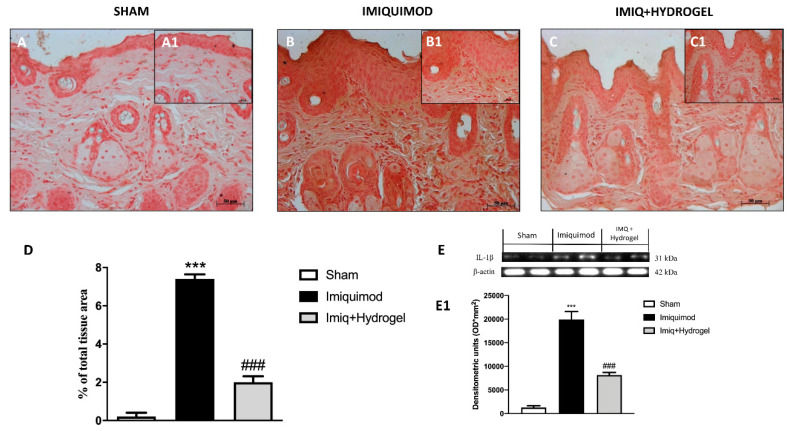
Effect of Hydrogel treatment on IL-1β expression. Immunohistochemical analysis of IL-1β demonstrated a considerably increased expression in the vehicle group (**B** magnification 20×, **B1** magnification 40×, **D**,**E**,**E1**) compared to the control group (**A** magnification 20×, **A1** magnification 40×, **D**,**E**,**E1**). Hydrogel treatment reduced the expression of IL-1β in animals subjected to IMQ damage (**C** magnification 20×, **C1** magnification 40×, **D**,**E**,**E1**). Data are representative of at least three independent experiments; One-Way ANOVA test. *** *p* < 0.001 vs. sham; ^###^ *p* < 0.001 vs. IMQ.

The key role of TNF-α and IL-1β as cytokines involved in the pathogenesis of psoriasis is well known [22,23]. Given the above evidence, we analyzed, through immunohistochemical staining and Western blot analysis, the expressions of TNF-α and Il-1β, cytokines that have a fundamental role in promoting the inflammatory cascade in many diseases. No expressions of both proteins were observed in the sham group (Figure 6A, A1, see histological score Figure 6D and Figure 7A, A1, see histological score Figure 7D). In the IMQ-treated group, the expressions of both TNF-α (Figure 6B, B1, see histological score Figure 6D) and Il-1β (Figure 7B, B1, see histological score Figure 7D), were significantly increased highlighting an inflammatory state due to IMQ-induced psoriasis-like dermatitis. CALIX/CUR hydrogel treatment caused downregulation of both pro-inflammatory cytokines, indicating a good ability to moderate the inflammatory state (Figure 6C, C1, see histological score Figure 6D and Figure 7C, C1, see histological score Figure 7D). We confirmed the data obtained for TNF-α and Il-1β by immunohistochemical, also with Western blot analysis (Figure 6E and densitometric analysis Figure 6E1; Figure 7E and densitometric analysis Figure 7E1).

### 2.6. Effect of Hydrogel Treatment on iNOS

**Figure 8:**
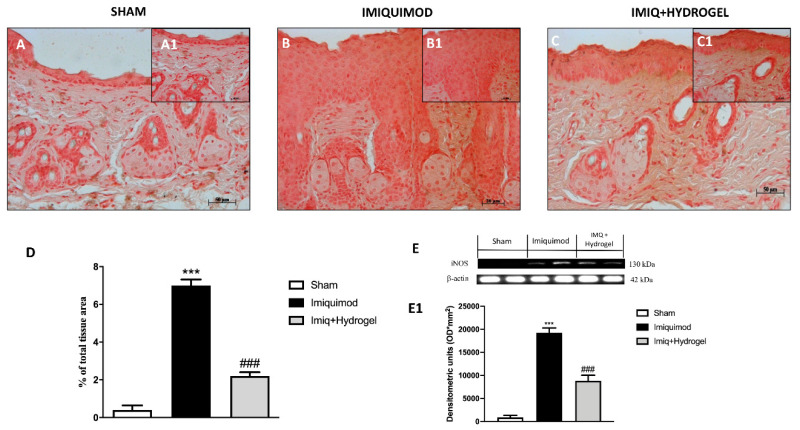
Effect of Hydrogel treatment on iNOS levels. By immunohistochemical analysis, it turned out that the level of iNOS was increased in mice of vehicle groups that received IMQ treatment (**B** magnification 20×, **B1** magnification 40×, **D**,**E**,**E1**) compared to the sham-operated mice (**A** magnification 20×, **A1** magnification 40×, **D**,**E,E1**). Moreover, Hydrogel treatment significantly decreased iNOS levels (**C** magnification 20×, **C1** magnification 40×, **D**,**E**,**E1**). Data are representative of at least three independent experiments; One-Way ANOVA test. *** *p* < 0.001 vs. sham; ^###^ *p* < 0.001 vs. IMQ.

The inflammatory state was accompanied by nitrosative stress with the upregulation of ROS generating enzymes [24]. Therefore, considering this cross-talk, we analyzed, by immunohistochemistry and Western blot, the enzyme iNOS, which represents the inducible form of nitric oxide synthetase. iNOS levels were not detected in the sham group (Figure 8A, A1, see histological score Figure 8D); while they were significantly increased in mice administered with IMQ (Figure 8B, B1, see histological score Figure 8D). Instead, CALIX/CUR hydrogel treatment downregulated iNOS levels, proving effective in moderating oxidative stress conditions (Figure 8C, C1, see histological score Figure 8D) associated with psoriasis. The data obtained for iNOS by immunohistochemical were also confirmed by Western blot analysis (Figure 8E and densitometric analysis Figure 8E1).

## 3. Discussion

Psoriasis, a T-cell-mediated inflammatory immune disease, is characterized by a thickened epidermis resulting from hyperproliferative keratinocytes and inflammatory leukocyte infiltration into the dermis and epidermis. Skin inflammation is one of the main hallmarks of psoriasis in which a complex of pro-inflammatory cytokines determines the degree and the pathogenesis of this disease [25]. Patients affected by psoriasis showed high levels of pro-inflammatory cytokines such as TNF-α, IL-6, and IL12 that exacerbate the inflammatory response [25,26]. Various topical and systemic treatments for psoriasis are commercially available, but still none of these are without side effects or able to act in short-term administration.

In the last decade, many researches have been performed to understand the role of CUR in skin diseases. Many are the evidence supporting its therapeutic efficacy [6]. The first one is that CUR, with its anti-inflammatory and antioxidant properties, can reduce inflammation caused by psoriatic plaques. Despite this, the main problem with CUR is that it has a fairly low solubility and poor skin penetration capacity [27]. These factors contribute to the reduced absorption of CUR (due to its limited accumulation within psoriatic lesions) but also to its rapid elimination from the epidermis. All this limits the therapeutic efficacy of CUR against psoriasis. Therefore, nanohydrogel systems, and the formulations that can be drawn from them, could represent a promising solution, favoring the use of CUR and reducing its limitations. In a previous paper, we reported that the CALIX entraps CUR through non-covalent interactions, improves the drug solubility of about 9000 times compared to CUR alone, and prevents the rapid degradation of CUR [16]. The CALIX/CUR colloidal solution administered as an eye drop resolved signs of LPS-induced uveitis in rats. In a subsequent work, we observed that under stimulus concentration CALIX and CUR form a supramolecular micellar nanohydrogel constituted by biofriendly components like the CALIX, CUR, and PBS at physiological pH; without the addition of additives or organic solvents. Rheological measurements showed that the CALIX/CUR hydrogel is viscoelastic, injectable, and self-healing. A qualitative analysis evidenced that the nanohydrogel is adhesive and absorbable by the skin. TEM and AFM images presented the tridimensional net of micellar nanoaggregates from which the hydrogel is formed. The hydrogel slowly dissolved in CUR-loaded micellar nanoaggregates, and the maintenance of the drug in the micelles appears advantageous in skin deposition and penetration because it allows a larger surface contact and avoids the rapid precipitation of the drug on the skin surface. As a nanohydrogel, the CALIX/CUR offers the advantages to combine the mechanical properties of a gel with the drug delivery properties of a micelle. Thus, in this study we evaluated the anti-psoriasis effect of the CALIX/CUR hydrogel by using IMQ-induced skin inflammation model that is currently the most widely accepted psoriasis animal model as it has many of the significant markers of human disease, including histopathology of lesions, specific cytokine expression patterns, and cellular infiltrates. Psoriasis disease is histologically characterized by hyperkeratosis, immune cell infiltration, parakeratosis, and neovascularization. The mice epidermis treated with CALIX/CUR hydrogel remained intact. Analogously to the CALIX/CUR colloidal solution applied on the ocular surface of mice, no inflammatory symptoms and no obvious change in histopathological features were observed in the treated skin, indicating that the hydrogel is safe for topical application. In normal epidermis, cells of the granular layer are interconnected by TJs that are cell-cell junctions that form paracellular barriers for solutes and inflammatory cells. The TJs include occludin, ZO-1, and claudin-1 and -4 structural proteins. Since it is known that inflammation is involved in the alterations of TJ proteins, we detected the profile of occluding and ZO-1 in the psoriasis model. Aberrant keratinocyte differentiation is linked with changes in the expression and distribution of TJ components. In the psoriasis plaques, where the horny layer is missing or abnormal, occludin and ZO-1 are found in more layers than in normal epidermis. Particularly, occludin and ZO-1 are revealed in a broader zone, ranging from the granular layer to the middle spinous layer, concentrated in the lateral and in the entire plasma membranes, although they can also be observed in the cytoplasm. The scientific world has highlighted the importance of restoring the normal occludin and ZO-1 distribution, to reduce the keratinization process clinically. The treatment with the CALIX/CUR hydrogel reduced the expression of occludin and ZO-1 that resumed a normal-looking profile, being restricted to the upper epidermis only. Moreover, it has well known that the immune system is strongly implicated in the pathogenesis of psoriasis, including both acquired immunity via T cells, and innate immunity, involving neutrophils, mast cells and keratinocytes. In psoriasis lesions, the number of T cells and dendritic cells (DCs) [28] is markedly increased [29]. T cells and DC infiltrate the epidermis migrating into all layers of the epidermis [30] and as a consequence levels of the pro-inflammatory cytokines IFN-α, IFN-γ, TNF-α, IL-1β, IL-23, and IL-17 are high in psoriasis lesions [31,32].

The topical application of IMQ, on mouse skin, can induce psoriasis lesions by alteration of the IL-23/IL-17 axis. IMQ application on mouse skin results in the rapid proliferation of DCs and stimulates mast cells to increase cytokine production [33].

TNF-α is an important cytokine in psoriasis, it plays a multi-faceted role [34]. TNF-α mediates immune response, inflammation, and apoptosis [35]. Since TNF-α is a central point in psoriasis pathogenesis, [26] anti TNF-α therapies [26,36] have proven a successful treatment. CUR has multiple pathways through which can effectively treat psoriasis. Among them, the capability to block both the production and action of TNF-α and reverse the anti-apoptotic function of TNF-α in skin cells is relevant. CUR has been reported to inhibit NF-κB and MAPK pathways and the expression of TNF-α induced IL-1β, IL-6, TNF-α and cyclin E. In our study, the levels of TNF-α and IL-1β were determined by immunohistochemical staining. The positive staining for both TNF-α and IL-1β was increased in the IMQ group in comparison to the normal group, indicating high expression of pro-inflammatory cytokines in psoriasis plaque. In comparison to the IMQ group, the application of CALIX-CUR hydrogel significantly reduced TNF-α and IL-1β expression highlighting that CUR incorporated in the calixarene-based hydrogel keeps its ability to suppress pro-inflammatory cytokines production.

The microvascular system suffers from the pathological changes associated with psoriasis; specifically, the local microvascular alterations result in dilation and tortuosity of capillaries, augmented permeability, and increase in endothelial vein formation, usually observed in lymph nodes. Many of the cells involved in inflammatory skin alterations are capable of releasing factors that act on the vascular system. The most important of these factors might be nitric oxide (NO), a well-established inflammatory agent produced by the activation of iNOS in inflammatory DCs [37]. In our study, we confirmed that iNOS is absent from normal skin, but it is significantly upregulated in psoriatic lesioned skin, focally in keratinocytes but to the greatest extent in the papillary dermis. CALIX/CUR hydrogel treatment reducing the expression of iNOS limited NO stimulates vessel dilation and permeability, acting as anti-vascular strategies. Although there are limitations and challenges with animal models and, in particular, with the in vivo model of psoriasis, this study has investigated an appreciated biological mechanism that clearly indicates the relationship between inflammation and psoriasis plaque formation. In particular, our findings suggest that sustained inflammatory processes can lead to psoriasis lesions.

Thus, clinically relevant models are essential for a fuller understanding of how inflammation of the numerous complex histopathological cascades in psoriasis lesion.

## 4. Materials and Methods

### 4.1. Preparation of the CALIX/CUR Hydrogel

The CALIX/CUR hydrogel was prepared, as reported in a previous paper [17]. In brief, 20 mg of choline-calixarene **1** [18] was dissolved in 1 mL PBS (10 mM, pH 7.4 Calbiochem, Sigma-Aldrich, Milan, Italy), then 1.8 mg of CUR (Sigma-Aldrich, Milan, Italy)was added.

The mixture was sonicated for 15 min, then stirred (500 rpm) at 37 °C up to complete dissolution of the CUR. The dose of CUR in the nanohydrogel corresponds to the maximum CALIX loading capacity [17].

### 4.2. Animals

Six-to 8-week-old, male BALB/c mice (ENVIGO, Milan, Italy) were housed in a controlled environment (22 ± 2 °C, 55 ± 15% relative humidity, 12 h light/dark cycle). The animals were acclimatized to their environment for 1 week and had ad libitum access to tap water and rodent standard diet. This study was approved by the University of Messina Review Board for the care of animals, in compliance with Italian regulations on protection of animals (n° 549/2018-PR released on 23 February 2018). Animal experiments were in compliance with Italian regulations on the protection of animals used for experimental and other scientific purposes (DM 116192) as well as EU regulations (OJ of EC L 358/1 18 December 1986).

### 4.3. Anti-Psoriatic Efficacy: IMQ Induced Psoriatic Plaque Model

Psoriasis-like lesions were induced by a daily topical application of a commercially available IMQ cream (62.5 mg/day for 7 days) on the shaved back skin of BALB/c mice, as previously reported [38,39] for 7 consecutive days. Animals were randomly assigned to the different groups: Sham psoriasis animals that were administered with Vaseline cream; IMQ animals, only challenged with imiquimod cream, and IMQ animals treated with hydrogel that was topically applied using a nylon mesh for even distribution of the hydrogel, for the whole the duration of the experiment. On day 7, in the 4 h following the last IMQ or Vaseline administration, the animals were sacrificed and the skin samples were taken. After which the samples were processed for histological and immunohistochemical analyzes.

### 4.4. Experimental Groups

The mice were randomly divided into 3 groups, as described below:

Group 1: Sham + vehicle: Mice received Vaseline cream without IMQ treatment for 7 days (*n* = 8);

Group 2: IMQ + vehicle: Mice received vehicle (PBS) with IMQ treatment for 7 days (*n* = 8);

Group 3: IMQ + Hydrogel: Mice were administered topically with Hydrogel daily for 7 consecutive days. (*n* = 8).

When the experiment was ended, the animals were sacrificed on the 7th day and euthanized by sevoflurane overdose, and the skin was removed for histological and immunohistochemical analysis.

The minimum number of mice for every technique was estimated with the statistical test a priori power studies with the G-power software, this statistical test supplied a professional method to analyze the sample size required to do the experiments.

### 4.5. Histological Analysis

To detect epidermal thickness, infiltration of inflammatory cells, and morphological variations, sections were stained with hematoxylin and eosin staining (H&E, Bio-Optica, Milano, Italy). Histological analyses were performed as previously described [40]. Portions of dorsal skin samples were quickly removed and fixed with 10% buffered formalin for at least 24 h at room temperature. After dehydration in graded ethanol and xylene, samples were embedded in paraffin and sectioned at 7 μm thickness. After the staining procedure, sections were observed by an optical microscope (Axostar Plus equipped with Axio-Cam MRc, Zeiss). The histological results were shown at 10× (100 μm of the Bar scale). All the histological analyses were executed in a blinded manner.

### 4.6. Toluidine Blue Staining

To assess mast cell quantity and their degranulation, epidermal sections were stained with toluidine blue (Bio-Optica, Milano, Italy). Sections were stained blue, highlighting mast cells that stained purple. The number of metachromatic stained mast cells was obtained by counting 5 high- power fields (40×) for the section using an Axiovision Zeiss (Milan, Italy) microscope and this correlated AxioVision software (Carl Zeiss Vision, Jena, Germany). Data were reported as the mean with standard deviation (SD). Toluidine blue staining results were shown at 40× magnification (20 µm scale bar).

### 4.7. Immunohistochemical Localization of ZO-1, Occludin, TNF-α, IL-1β and NOS2

Immunohistochemical localization was made, as previously described by Esposito et al. [40]. Slices were incubated at room temperature overnight with one of the following primary antibodies: Anti-ZO1 (617300 Invitrogen, 1:200 in PBS, *v/v*), anti-occludin (71-1500 Invitrogen, 1:200 in PBS, *v/v*), anti-TNF-α (sc-52746 Santa Cruz Biotechnology, 1:100 in PBS, *v/v*) anti-IL-1β (sc-32294 Santa Cruz Biotechnology, 1:50 in PBS, *v/v*) and anti-iNOS (610432 BD Transduction, 1:50 in PBS, *v/v*). At the end of the incubation with the primary antibody, the sections were washed with PBS and incubated with a secondary antibody (Santa Cruz Biotechnology, Santa Cruz, CA, USA) for 1 h. The reaction was revealed by a chromogenic substrate (brown DAB), and counterstaining with NUCLEAR FAST-RED. A negative control was performed using no primary antibody, particularly, tissue was incubated with the antibody diluent alone, followed by incubation with secondary antibodies and detection reagents.

All stained sections were observed and analyzed as previously described. For immunohistochemistry 20× (50 µm scale bar) and 40× (20 µm scale bar) were shown.

### 4.8. Western Blot Analysis

The levels of TNF-α, Il-1β and iNOS were quantified in skin samples collected 7 days after IMQ induce psoriasis. Cytosolic proteins were prepared and separated electrophoretically to be transferred to nitrocellulose membranes as previously described [41]. Membranes were blocked with 5% (*w/v*) non-fat dried milk in buffered saline (PM) for 45 min at room temperature and subsequently probed with specific antibodies: anti- TNF-α (1:500; Santa Cruz Biotechnology #sc-52746), anti-Il-1β (1:500; Santa Cruz Biotechnology #sc-32294) and anti- iNOS (1:500; 610432 BD Transduction) in 1× PBS, 5% *w/v* nonfat dried milk, and 0.1% Tween-20 (PMT) at 4 °C overnight. Membranes were incubated with peroxidase-conjugated bovine anti mouse immunoglobulin G (IgG) secondary antibody or peroxidase-conjugated goat anti-rabbit IgG (1:2000, Jackson ImmunoResearch, West Grove, PA) for 1 h at room temperature. To establish that blots were loaded with equal amounts of proteins, they were also incubated in the presence of the antibody against β-actin protein (1:500; Santa Cruz Biotechnology #sc-8432). Signals were revealed with enhanced chemiluminescence (ECL) detection system reagent according to the manufacturer’s instructions (Thermo, USA). The relative expression of the protein bands was quantified by densitometry with BIORAD ChemiDocTMXRS+software and standardized to β-actin levels, as an internal control.

### 4.9. Materials

BALB/c (6-week-old, male) mice were purchased from Envigo (Udine, Italy); IMQ topical cream was purchased from Aldara^®^ 5% cream, Meda AB, Solna, Sweden; Sevofluorane 100% was purchase from Baxter (Rome, Italy); 10% neutral buffered formalin was obtained from Bio Optica (Milan, Italy); absolute ethanol was purchased from Carlo Erba (Milan, Italy); Hematoxylin and eosin were purchased from Bio Optica (Milan, Italy). All other chemicals were obtained from commercial sources and were of the highest grade accessible. All stock solutions were made in non-pyrogenic saline (0.9% NaCl, Baxter, Milan, Italy).

### 4.10. Statistical Analysis

The values of each result were displayed as the mean ± standard error of the mean (SEM) of N observations, where N represents the number of animals used.

The whole experiment was representative of at least three experiments performed on sections of animals belonging to the various experimental groups. These experiments were conducted on different days.

The data were collected and subsequently analyzed by one-way ANOVA followed by a post-hoc Bonferroni test for multiple comparisons. A *p*-value of less than 0.05 was considered significant.

## 5. Conclusions

In summary, for the first time a nanohydrogel built by a calix[4]arene macrocycle has been investigated as a skin drug delivery system. The nanohydrogel formed by the micellar CALIX and CUR exhibited no significant toxicity and effective anti-psoriatic effect in an IMQ-induced psoriasis model, by reducing the pro-inflammatory process. Our results evidenced that CUR keeps its anti-inflammatory activity when entrapped in the calixarene-based hydrogel and corroborated that the nanohydrogel due to its capacity to solubilize and preserve CUR from rapid degradation. Its mechanical properties such as skin spreadability, adhesivity and penetration, and its ability to slow drug release can be a new tool for the delivery of CUR in the skin and a new anti-psoriatic approach that could better derive patient satisfaction, improving efficacy and comfort compared to canonical treatments.

## Abbreviations

CUR	curcumin
CALIX	micellar choline-calix[4]arene amphiphile
IMQ	imiquimod
TJs	tight junctions
DCs	dendritic cells
NO	nitric oxide
iNOS	inducible nitric oxide synthetase
